# Meningococcic Carriers

**Published:** 1916-06

**Authors:** 


					﻿Meningococcic Carriers. Sidney T. Champtaloup and John T. Bowie, Bacteriologic I)ept., Otago Univ., New Zealand, N. Z. Med. Jour., Feb., present the following tables showing positive results with cultures from the naso-pharynx:
OUR TABLE.	BRUNS & HOHN’S TABLE.
Carriers.	Carriers.
From 0- 1 week 1 case	Up to 8 days 28
11	1-2	weeks	2	cases	11	2	weeks	18
“	2-	3	“	3	“	11	3	“	13
“	3- 4	•	“	1	case	“	4	“	10
“	4- 5	“	2	cases	“	5	“	4
“	5-	6	“	2	“	“6	“	3
“	6-	7	“	4	“	“	rj	“	3
“	7-	8	“	0	11	“	8	“	1
“	8-	9	“	9	“	11	11	“	1
“	9-10	“	3	11
27	81
				

